# The expansive effects of polyamines on the metabolism and virulence of *Streptococcus pneumoniae*

**DOI:** 10.1186/s41479-021-00082-x

**Published:** 2021-03-25

**Authors:** Bindu Nanduri, Edwin Swiatlo

**Affiliations:** 1 Department of Comparative Biomedical Sciences, College of Veterinary Medicine, Mississippi State University, MS 39762 Mississippi State, USA; 2 Institute for Genomics, Biocomputing and Biotechnology, Mississippi State University, Mississippi State, MS 39762 USA; 3 Section of Infectious Diseases, Southeast Louisiana Veterans Health Care System, New Orleans, LA 70112 USA

**Keywords:** Pneumococci, Polyamine, Capsule, PotD, Immunization, Virulence, Stress response, Autolysis

## Abstract

Polyamines are common intracellular metabolites of nearly all cells, and their conservation across a vast diversity of cells suggests critical roles for these compounds in cellular physiology. Most intracellular polyamines are associated with RNA and, subsequently, polyamines have significant effects on transcription and translation. Putrescine and spermidine are the most common polyamines in bacteria. Intracellular polyamine pools in bacteria are tightly controlled by both de novo synthesis and transport. Polyamine homeostasis is emerging as a critical parameter of multiple pathways and physiology with substantial impact on bacterial pathogenesis, including the important human pathogen *Streptococcus pneumoniae*. Modulation of polyamine metabolism in pneumococci is an important regulator of central metabolism. It has broad effects on virulence factors such as capsule as well as stress responses that ultimately impact the survival of pneumococcus in a host. Polyamine transport protein as a single antigen or in combination with other pneumococcal proteins is shown to be an efficacious immunogen that protects against nasopharyngeal colonization, and invasive disease. A comprehensive description of polyamine metabolic pathways and their intersection with pneumococcal pathogenesis will undoubtedly point to novel approaches for treatment and prevention of pneumococcal disease.

## Background

Lower respiratory tract infections continue to be an important cause of morbidity and mortality globally and are responsible for significant health care expenditures, particularly in developing countries with limited resources available for health care [1, 2]. World-wide, pneumonia is the leading cause of death of children under 5 years of age [3], and the fourth most common cause of death across all age groups [4]. The microbiological diagnosis of pneumonia is difficult under the best circumstances and is even more formidable in resource-limited settings. However, most systematic studies of pneumonia identify *Streptococcus pneumoniae* (pneumococcus) as the most common bacterial etiology across all age groups [1, 5–9]. At the present time vaccine development and deployment have significantly reduced invasive infections in those countries where uptake is highest [3, 10]. Nevertheless, current vaccines are based on polysaccharides comprising the capsules associated with most virulent pneumococcal strains, and there are currently 100 distinguishable pneumococcal serotypes [11]. It is clear that vaccines will be hard-pressed to remain current with the serotypes causing disease in widely dispersed geographical areas, as migrating populations and serotype replacement under immune pressure constantly change the endemic serotype landscape [12–14]. Additionally, treatment of pneumococcal infections, much like many other bacterial infections, is becoming more complex and expensive because of the continuing emergence of antibiotic-resistant strains [15–18].

Novel targets for antibacterial drugs and invariant antigens constant across all pneumococcal serotypes are critical to reducing the burden of pneumococcal disease. Human bacterial pathogens, particularly opportunists such as pneumococci that normally live a commensal lifestyle, must adapt to survive and thrive at disparate anatomic sites. Each site presents markedly different environmental stimuli and stress to the bacterial cells. It is clear that pneumococci regulate central metabolism in response to different micro-environments of the host, not just a small sub-group of genes coding for well-studied virulence factors. Identification of metabolic pathways and regulatory mechanisms critical for environmental adaptation will point to new targets for intervention and prevention of pneumococcal infections.

### Polyamines

**Fig. 1:**
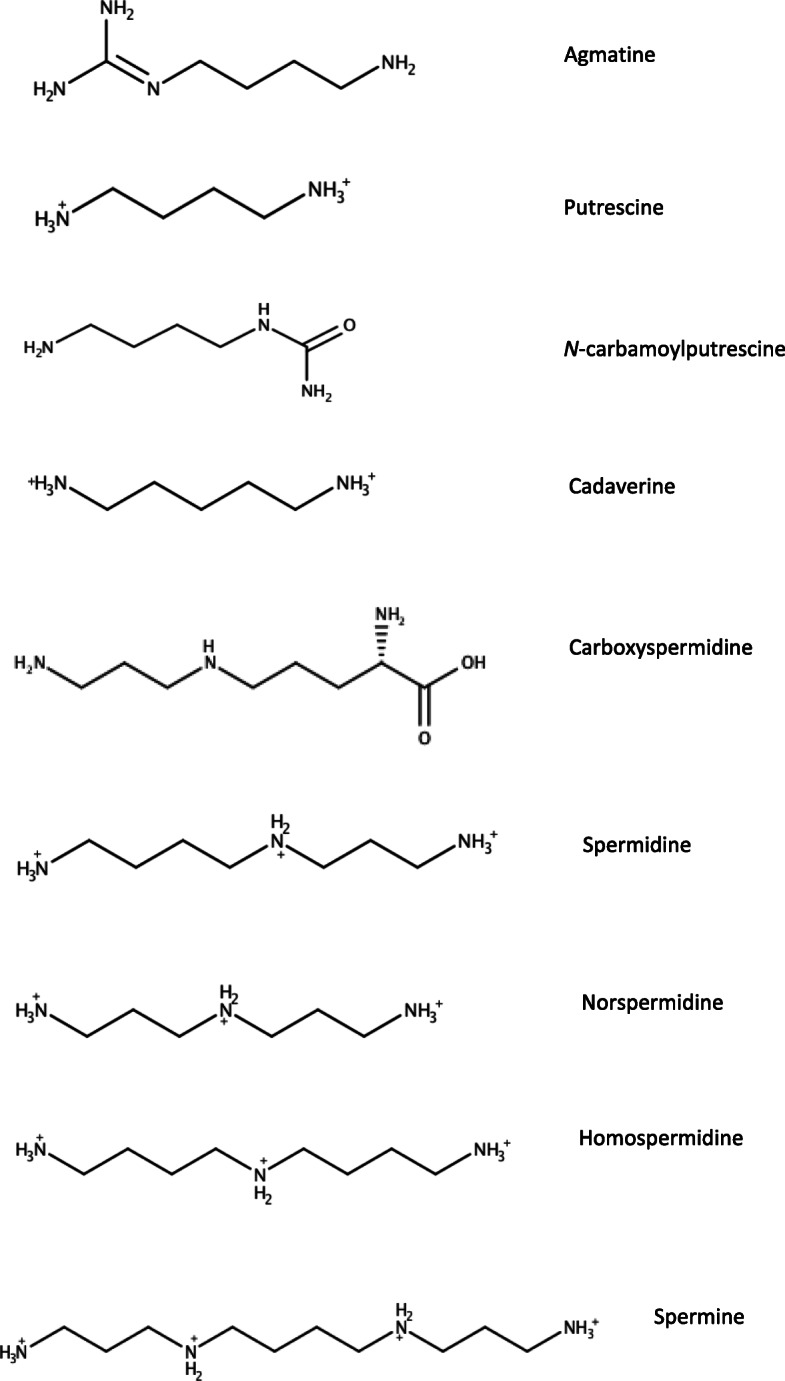
Structures of polyamines most commonly found in prokaryotes

Polyamines are small molecules consisting of a hydrocarbon backbone with multiple interspersed amino groups. They are found in most cells in all domains of life and are intimately involved in a wide variety of cellular functions. There are many different types of polyamines in cells (Fig. 1) but putrescine and spermidine are the most common in bacteria. Agmatine and cadaverine generally occur in lower concentrations and spermine is distinctly rare in most bacterial species [19]. Polyamines have been associated with a broad variety of cellular processes but the most consequential association is between polyamines and nucleic acids, primarily RNA [20]. Most intracellular polyamines are associated with RNA and, subsequently, polyamines have significant effects on transcription and translation [21–24]. Polyamines occur in all bacterial phyla but the remainder of this introductory section will focus on bacteria that are frequently associated with human infections.

**Fig. 2:**
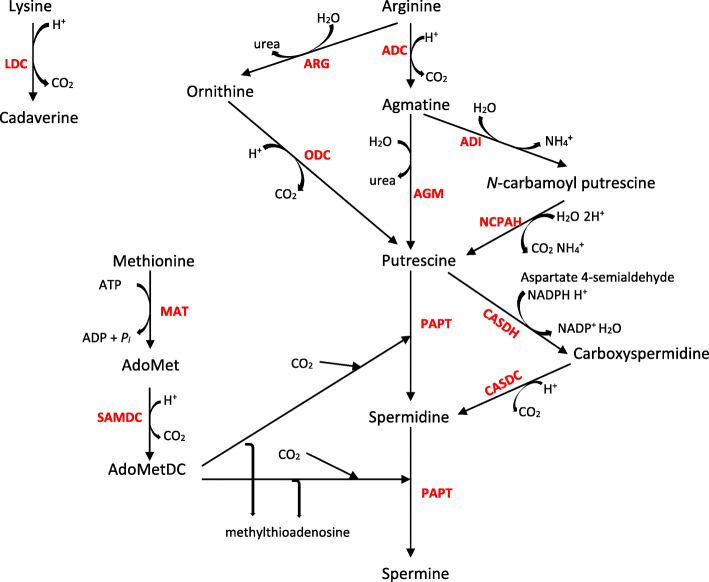
Central common polyamine synthesis pathways in prokaryotes. ADC – arginine decarboxylase, ARG – arginase, ODC – ornithine decarboxylase, AGM – agmatinase, ADI – agmatine deiminase, NCPAH – N-carbamoylputrescine amidohydrolase, CASDH – carboxyspermidine dehydrogenase, CASDC – carboxyspermidine decarboxylase, PAPT – polyamine aminopropyltransferase, MAT – methionine adenosyltransferase, SAMDC – S-adenosylmethionine decarboxylase, LDC – lysine decarboxylase; AdoMet – S-adenosylmethionine, AdoMetDC – decarboxylated S-adenosylmethionine

Intracellular polyamine pools in bacteria are generated primarily by synthesis from decarboxylation of amino acids arginine, ornithine, and lysine (Fig. 2). In addition to biosynthesis many human pathogenic bacteria have putative polyamine transporters for uptake from extracellular environment [19]. The polyamine transporters of *Escherichia coli* have been the most extensively studied, but most human bacterial pathogens have annotated transporters, including pneumococcus [19, 25–27]. The contribution of these potential membrane transporters to intracellular polyamine homeostasis and cellular physiology is mostly unstudied and poorly understood in most pathogenic bacteria.

A broad array of roles has been ascribed to polyamines in bacterial cells but their effects on transcription and translation are probably paramount [24, 28, 29]. This paradigm was primarily developed from work done with *E. coli* but it is true for many eukaryotic cells and likely true for most bacterial taxa as well. The polycationic structure of polyamines naturally predicts interactions with polyanionic molecules such as nucleic acids. There are some reports that describe growth-promoting activity of polyamines in bacteria, which may reflect the positive effects of polyamines on protein synthesis. Polyamine synthesis is essential for growth of *Pseudomonas aeruginosa* [30] and *Campylobacter jejuni* [31]. While there are no consensus polyamine biosynthesis genes in *Borrelia burgdorferi*, this pathogen does contain an annotated polyamine transporter, PotABCD, which is necessary for cell growth [32]. This transporter, and by implication intracellular polyamines levels, also effects global gene expression and the antigenic structure of *B. burgdorferi* [33]. In contrast to these examples, some important human pathogens do not have any discernible requirement for polyamines. *Staphylococcus aureus* and *Enterococcus faecalis* do not contain any intracellular polyamines and growth is unaffected by exogenous polyamines. In fact, extracellular polyamines at physiological levels are toxic to *S. aureus* cells [34, 35].

### Polyamines and human bacterial pathogens

Other than *E. coli* as a model system for polyamine metabolism in bacteria (reviewed in [36]) the role of these molecules in pathogenesis has received comparatively little attention. The respiratory pathogen *Legionella pneumophila* resides in intracellular vacuoles and requires polyamines to replicate optimally in cultured human macrophages [37]. Deletion of a *potD* homologue in *L. pneumophila* results in a variety of seemingly unrelated phenotypes including Na^+^ hypersensitivity, loss of host-cell attachment, and vacuole trafficking defects [38]. This is not entirely unexpected considering the pleiotropic effects of polyamines on gene expression. *Neisseria gonorrhoeae* expresses a membrane transporter for spermidine/spermine but intracellular polyamines have not been linked to specific phenotypes [39]. The ability of *Proteus mirabilis* to swarm is critical for colonizing the urinary tract in the presence of urethral catheters. This swarming trait is controlled by a large number of genes but putrescine synthesis is one capacity essential for *Proteus* differentiation into swarmers [40–44].

The formation and disassembly of biofilms are complex traits which are attributed to many gene products. For many pathogenic bacteria polyamines promote biofilm synthesis and maturation. In *Vibrio cholerae* both spermidine and norspermidine can bind a PotD homologue in the periplasmic space and effect up-regulation of biofilm production [45–47]. Putrescine synthesis by decarboxylation of arginine positively correlates with biofilm production in *Yersinia pestis*, the etiology of human plague [48]. Agmatine is sensed by *Pseudomonas aeruginosa* which in response up-regulates transcription of agmatine deiminase and subsequently increases intracellular putrescine concentration. Like *Yersinia*, increased putrescine levels in *P. aeruginosa* correlate with increased biofilm production [49]. In contrast to these examples, polyamines are inhibitory to biofilm production in *Neisseria gonorrhoeae* [50] and *Staphylococcus epidermidis* [51]. Interestingly, polyamines are dispensable for growth and have no effect on biofilms in *Staphylococcus aureus* [34].

Bacteria encounter myriad host responses which seek to constrain microbial multiplication and distant translocation at both mucosal surfaces and deeper anatomic sites. These host responses entail a variety of chemical defenses, but a large component of these defenses involve reactive oxygen and nitrogen radicals as well as acid generation. Because of their polycationic nature, polyamines are efficient scavengers of both oxygen- and nitrogen-based radicals [52–54]. Additionally, polyamines can regulate the expression of genes which subsequently leads to enzymatic detoxification of reactive oxygen or nitrogen compounds [54–56]. In *P. aeruginosa* endogenously synthesized polyamines bind to the outer membrane and confer resistance to oxidative stress as well as certain antibiotics [57]. In *E. coli* dynamic regulation of polyamine metabolism has been associated with severe osmotic shifts [58], high temperature [59], and rapid pH shifts [60].

Polyamines are found in cells ranging from the smallest and simplest prokaryotes to complex vertebrates. This conservation suggests that polyamines fulfill critical functions essential to the most common metabolic pathways among free-living organisms. Polyamine metabolism and its effects on cells are poorly understood and this is certainly no less true for bacterial pathogens. The remainder of this review will bring together what is known about polyamines in pneumococci and how polyamines effect physiology and the natural history of pneumococcal infections.

#### *Streptococcus pneumoniae* and polyamines

##### Polyamine biosynthesis and transport genes in pneumococcal genomes

Genes involved in polyamine metabolism are well characterized in *E. coli* (Fig. 2) and serve as a scaffold for identifying homologs in other sequenced bacterial genomes, including pneumococcus. Sequence alignment and analyses showed that *speE* encodes a polyamine aminopropyltransferase (PAPT) that can synthesize spermidine from putrescine and decarboxylated S-adenosylmethionine, and spermine from spermidine. PAPT (*speE*) has the characteristic glycine-rich aminopropyltransferase motif [61]. Unlike Gram-negative bacteria, spermidine biosynthesis in pneumococci does not involve S-adenosylmethionine decarboxylase, encoded by *speD* [62], which is not annotated in the genome. In a two-step reaction catalyzed sequentially by carboxyspermidine dehydrogenase and carboxyspermidine decarboxylase, spermidine is synthesized from putrescine [63]. While the initial annotation identified a putative lysine decarboxylase that catalyzes the synthesis of cadaverine, current annotation of pneumococcal genomes indicates that this gene is an arginine decarboxylase (*speA*) that synthesizes agmatine from arginine in the putrescine biosynthesis pathway. Comparison of the  kinetics of the enzymatic conversion of arginine, lysine and ornithine substrates by recombinant SP0916 confirms that arginine is the preferred substrate for this decarboxylase. Thus SP0916 gene in serotype 4 is indeed an arginine decarboxylase (*speA*) [64]. Thus, pneumococcal genomes lack annotated cadaverine biosynthesis gene *cadA*. The old annotation (*cadA,* lysine decarboxylase) from literature will be replaced with the current annotation (*speA*, arginine decarboxylase) in the following sections.

Polyamine uptake in pneumococcus is predicted to be via a single ATP-binding cassette (ABC) transporter, organized as a four-gene operon denoted *potABCD*. Based on homology with *E.coli* genes, the pneumococcal transport operon is predicted to transport putrescine and spermidine. The proposed structure of putrescine/spermidine transporter predicts that PotD is a substrate-binding lipoprotein, PotBC form transmembrane channels that transport polyamines, and PotA is a membrane-associated cytosolic ATPase [65]. Transcriptional analysis of *potABCD* in WU2 identified that it is co-transcribed with *murB*, as a part of a polycistronic mRNA with [66]. Since *murB* encodes UDP-N-acetylenolpyruvoyl glucosamine reductase, an enzyme involved in peptidoglycan biosynthesis, it is likely that polyamine transport is intricately linked with the regulation of structural components, such as peptidoglycan in pneumococci. Both polyamines and peptidoglycan synthesis are critical for rapidly dividing cells, so it is not entirely surprising that there is some degree of co-regulation at the level of transcription.

Using high-titer polyclonal antiserum against recombinant PotD, it was determined that PotD is accessible to antibodies at the surface of intact bacteria, in both unencapsulated and highly hydrated and mucoid capsule type 3 [27]. PotD is primarily associated with cytoplasmic membranes, corroborating functional genomic analysis that it is a lipoprotein. Immunoblot assay with whole cell lysates indicate that PotD is antigenically conserved among diverse pneumococcal capsule types such as 2, 3, 4, 6A, 9, 10, 14, 19, and 23 [61].

PCR analyses suggest that *speA, speE* and *potD* are conserved among capsular serotypes of *S. pneumoniae* commonly associated with invasive disease [61]. Furthermore, BLAST analyses with *speA, speE,* and *potABCD* sequences showed conservation of these genes with more than 99% identity in the genomes of all sequenced pneumococcal isolates. The current annotation of polyamine metabolism in pneumococcal genomes demonstrates some notable absences. For example, a key biosynthesis enzyme of putrescine biosynthesis, ornithine decarboxylase (ODC), that catalyzes the conversion of ornithine to putrescine, is not annotated in publicly available pneumococcal genomes. Polyamine acetyltransferases that catalyze sequestration of free polyamines by acetylation, and deacetylases that use acetylated polyamine substrates to release free polyamines are also not described.

### Polyamines and pneumococcal growth

Pneumococci require either choline or ethanolamine for growth. Comparison of the growth kinetics of WU2 in chemically defined medium (CDM) supplemented with choline, ethanolamine, and putrescine identified no differences in the rate of cell division during exponential growth. However, amongst all three compounds, putrescine resulted in the longest delay before the onset of exponential growth. Binding with choline is required for activation of the enzymatic activity of pneumococcal autolysin LytB, responsible for daughter cell separation during replication. Growth in polyamine supplemented CDM resulted in an elongated chain phenotype that is similar in morphology to an autolysin-deficient strain [66]. Although putrescine associated with the cell wall at levels comparable to that of choline and ethanolamine, it could not bind and anchor choline-binding proteins [66].

Deletion of *potD* in WU2 does not impact in vitro growth in CDM that does not contain exogenous polyamines. Polyamine biosynthesis inhibitors such as difluoromethyl ornithine that inhibits ODC and methylgloxal-bis (guanyl hydrazone), an inhibitor of adenosylmethionine decarboxylase, delay growth of WU2*ΔpotD* [65]. Deletion of three genes involved in spermidine synthesis: carboxyspermidine dehydrogenase, arginine decarboxylase, and spermidine synthase in D39 did not impact growth in polyamine-free medium, indicating that polyamine synthesis is dispensable for growth [63]. However, with all three deletion strains, there was a significant delay in the onset of autolysis, possibly by modulation of the activity of autolysin LytA by spermidine [63]. Growth of *S. pneumoniae* TIGR4 *ΔspeA, ΔspeE* and *ΔpotABCD* strains in Todd-Hewitt yeast extract (THY) complete medium was comparable to that of the wild-type (WT) [61], although there was a significantly longer lag phase in *ΔspeA* [67].

### Measurement of intracellular polyamines

Measurement of intracellular polyamines indicates that spermidine is the most abundant polyamine in type 3 strain followed by putrescine and cadaverine, when cultured in THY, a complete growth medium that contains polyamines or CDM that does not have polyamines [65]. Modulation of polyamine metabolic pathways either by gene deletion or chemical inhibition will impact the intracellular concentrations of polyamines. WU2 Δ*potD* had reduced levels of spermidine compared to WT in THY, but comparable levels in CDM supplemented with choline (Table 1). When cultured in the presence of polyamine synthesis inhibitors, increased levels of cadaverine were observed in *ΔpotD*. Addition of exogenous spermidine and putrescine restored growth of type 3 *ΔpotD* strain identical to that of WT growth. Taken together, these data suggest that alternate systems for synthesis and transport, yet to be described, exist in pneumococci [65]. In THY, intracellular concentration of spermidine > cadaverine > putrescine in serotype 4 strain (Table 1). Intracellular concentrations of all three polyamines were reduced when either synthesis or transport genes were deleted in type 4 strain [61]. Deletion of the polyamine transport operon (*potABCD*), predicted to transport putrescine and spermidine resulted in significant reduction of these two substrates in serotype 4 strain [64]. There was a significant reduction in the levels of agmatine, and N-acetylpsermidine in ∆*potABCD* [64]. Deletion of genes from spermidine synthesis pathway resulted in reduced spermidine levels in serotype 2 strain cultured in CDM [63], although there was no significant reduction when cultured in THY [64]. Arginine decarboxylase deficient type 4 pneumococci had significantly lower levels of agmatine, an intermediate in putrescine biosynthesis pathway [64]. In vitro studies utilizing complete growth medium that mimics the host environment i.e. a source of different polyamines, can enable the identification of polyamine metabolic pathways relevant to the phenotype in vivo. Studies with CDM that does not contain polyamines focus on polyamine synthesis pathways, without the compensation by transport. In vitro studies with pneumococci indicate that modulation of polyamine metabolic pathways alters intracellular polyamine levels, which could impact processes regulated by polyamines such as transcription and translation.

**Table 1:** Concentration of Intracellular polyamine in *S. pneumoniae* capsule type 3 (WU2, μM) and type 4 (TIGR4, pM) strains

Growth medium	Strin	Putrescine	Spermidine	Cadaverine
THY^a^	WU2	0.5 ± 0.04	25.6 ± 0.64	0.3 ± 0.05
CDM^b^	WU2	1.4 ± 0.08	19.0 ± 1.4	0.7 ± 0.12
THY^a^	TIGR4	4.1 ± 2.0	14.2 ± 1.2	9.2 ± 1.2

^a^Todd-Hewitt broth supplemented with yeast extract

^b^chemically defined medium

### Polyamines and stress responses

Transcription of *potD* is responsive to the availability of choline in the environment. Expression of *potD* is upregulated during choline deprivation and down-regulated in choline-rich conditions in serotype 2. Upregulation of *potD* under low choline conditions could indicate it’s involvement in binding and transport of choline [68]. The predicted substrate for the PotABCD transporter, putrescine, is structurally similar to choline and can substitute for choline during pneumococcal growth. Expression of *potD* increased in pneumococci exposed to hydrogen peroxide [68]. Exposure of WT and *speA, speE* and *potABCD* mutants to the oxidizing agent paraquat or transient exposure to low pH identified no significant differences in survival [61]. Expression of *potD* is sensitive to temperature. When exposed to either lower (34 °C) or higher (42 °C) temperatures relative to core human body temperature of 37 °C, expression of *potD* was upregulated [68].

### Role of polyamine transport and synthesis in virulence

Signature-tagged mutagenesis suggested a possible role for polyamine transport genes (*potA* and *potD*) [69], and arginine decarboxylase (*speA*/SP0916) [70] in pneumococcal pathogenesis. Strains harboring isogenic deletions in polyamine synthesis and transport genes have been invaluable tools to study the role of polyamine metabolism in pneumococcal virulence. Various murine models of infection indicated a role for PotD in virulence of a capsule type 3 [65]. Studies with deletions of *speA, speE* and *potABCD* in murine models of colonization, pneumonia and sepsis suggest that polyamine synthesis and transport contributes to pneumococcal virulence [61].

Polyamines are linked to pneumococcal virulence in encapsulated strains. In contrast to encapsulated strains, deletion of *potD* in nonencapsulated pneumococcus (NESp), has no impact on nasopharyngeal colonization, and translocation into the middle ear, in a mouse model [71]. Similar results with NESp were observed in murine models of pulmonary infection. In a chinchilla model of otitis media, deletion of *potD* in NESp did not result in a significant change in the number of bacteria recovered compared with the parent strain. However, significantly less bacteria were recovered from the middle ears of chinchillas infected with a capsule type 4 *potD* deletion compared to WT [71]. Polyamines appear to be important for virulence of encapsulated strains, however, they may have different functions and effects in naturally-occurring nonencapsulated strains.

### Polyamines and interactions with host

Impaired polyamine metabolic pathways alter intracellular concentrations of polyamines that results in changes in gene/protein expression that ultimately modulates survival of pneumococci in the host. In a murine model of pulmonary infection with type 4 WT and Δ*potABCD* [72] strains, significant differences in the in vivo growth were observed. Bacterial burden in the lung suggests that *ΔpotABCD* is more invasive but more susceptible to host defenses than WT, during early stages of infection. The cytokine/chemokine profile of lungs infected with *ΔpotABCD* showed significantly higher levels of G-CSF, LIF, IP-10, KC, GM-CSF, IL-5, IL-17 and MCP-1 consistent with the initial higher bacterial burden. Elevated levels of IL-17 correlate with the recruitment and activation of neutrophils for pneumococcal clearance during colonization [73]. There was a significantly higher infiltration of neutrophils in mice infected with *ΔpotABCD*. There was an increased uptake of *Δp*o*tABCD* by murine neutrophils that did not require opsonization, and *ΔpotABCD* is taken up more efficiently by murine alveolar macrophages relative to WT. Protein expression profile of lung infected with WT and *ΔpotABCD* indicated early activation of innate immune responses by *ΔpotABCD* that were delayed in the WT [72]. There is evidence for increased expression of polyamine transport in serotype 2 harvested from blood of bacteremic mice compared to growth in vitro [68]. Pneumococci regulate polyamine metabolism during growth in vivo, which alters their response to host and has implications for pathogenesis.

### Polyamines and regulation of gene/protein expression

Polyamine synthesis compensates for the loss of transport in serotype 4 strain [72]. However, deletion of putrescine synthesis results in reduced expression of transport genes and spermidine biosynthesis [67]. Impaired transport resulted in reduced expression of proteins that encode oligopeptide and amino acid ABC transporters involved in pathogenesis, and virulence factors such as capsular polysaccharide biosynthesis proteins, pneumolysin, pneumococcal surface protein A, and proteins involved in growth and replication in type 4 pneumococci. Proteomics analysis of *ΔspeE* indicated reduced expression of virulence factors such as oligopeptide and amino acid ABC transporters, zinc metalloprotease and choline-binding and cell division proteins, while expression of arginine decarboxylase was higher relative to type 4 WT strain. Polyamine mediated regulation of the expression of proteins involved in pneumococcal virulence could explain the reported attenuated phenotype of polyamine transport and synthesis impaired strains [61].

### Polyamine synthesis and capsule expression

The ability to regulate capsular polysaccharide (CPS) is critical for survival of pneumococci [11]. Colonization requires reduced CPS to expose adhesion molecules that interact with host cells while systemic infection requires a thick capsule to inhibit complement, prevent antibody deposition and resist opsonophagocytosis. Deletion of an arginine decarboxylase resulted in reduced CPS in serotype 4 strain [67] . Loss of capsule in Δ*speA* could be due to transcriptional control and metabolic re-programming [74] that limits availability of precursors for CPS. Thus, polyamine synthesis is critical for the production of CPS in pneumococci. Proteomics analysis of *ΔspeA*, identified reduced expression of proteins involved in peptidoglycan biosynthesis, oligopeptide ABC transporters, iron transporter, lysine biosynthesis, and higher expression phosphate transport, pentose phosphate pathway (PPP) and oxidative stress response proteins. This protein expression profile indicates a shift in central metabolism that favors PPP, which is often a hall mark of cellular response to increased oxidative stress. This shift in metabolism could limit the availability of precursors for CPS, which could explain the loss of capsule in this transport deficient type 4 strain [67].

Transcriptome analysis *ΔspeA* confirm the shift in central metabolism that favors PPP and identified gene expression changes that inhibit synthesis of nucleotide sugars. Untargeted metabolomics of arginine decarboxylase deficient pneumococci identified accumulation of metabolites that indicate inhibition of glycolytic activity [74], and depletion of compounds that can impact the ability to combat oxidative stress. Characterization of *ΔspeA* indicates reduced galactose to glucose interconversion via the Leloir pathway. This in turn will limit the availability of UDP-galactose, a precursor of serotype 4 CPS, and UDP-N-acetylglucosamine (UDP-GlcNAc), a nucleotide sugar precursor that is at the intersection of CPS and peptidoglycan repeat unit biosynthesis [74]. Reduced glycolytic activity and re-routing intermediates of glycolysis and upregulation of transketolases supports a shift in carbohydrate metabolism that favors PPP [74], at the expense of CPS synthesis.

Recent evidence indicates that deletion of polyamine transporter (*potABCD*) results in a nonencapsulated phenotype, while deletion of *speE* gene had no impact on CPS in serotype 4 [64]. Significantly reduced levels of agmatine correlate with reduced capsule in ∆*speA* and ∆*potABCD* strains, while levels of agmatine were comparable to that of wild type serotype 4 strain. Exogenous supplementation with agmatine restores CPS in both ∆*speA* and ∆*potABCD* strains [64]. Thus, agmatine is a critical regulator of CPS synthesis in pneumococci and inhibition of polyamine synthesis and supplementation with agmatine appear to be CPS OFF/ON switches that can be utilized to dissect the intersection between modulation of polyamine metabolism and CPS synthesis in Spn.

### Polyamines and NESp

In NESp that lack serological evidence of capsule expression [75], deletion of *potD* resulted in a significantly lower synthesis of pneumolysin, and reduced hemolytic potential compared to WT group II NESp [71]. Expression of *pspK*, that replaces the *cps* locus in group II NESp, was significantly higher at the protein and RNA levels, indicating that *potD* is a negative regulator of *pspK* in NESp. Adhesion of NESp Δ*potD* with A549 pulmonary epithelial cells was higher compared to the WT. Deletion of polyamine transport (*potD*) enhanced biofilm formation of NESp while it inhibited biofilm formation in encapsulated type 4 pneumococci [71].

### PotD as an immunogen

Given that deletion of PotD or PotABCD results in reduced virulence, PotD is conserved in multiple pneumococcal serotypes, and PotD has an extracellular domain, it was natural to examine the immunogenic potential of PotD. Active immunization of mice, and passive immunization of rabbits by PotD followed by systemic infection with type 3 strain showed that active immunization resulted in very-high-titer antibody responses, and better survival in mice. Passive immunization with rabbit antisera against PotD afforded protection against septicemia [76]. An optimal protein antigen would provide protection against both nasopharyngeal carriage as well as invasive disease. Mucosal immunization with PotD was evaluated for protection against colonization with a type 19F strain that does not easily cause invasive infection in mice [77, 78], and type 4 (that can invade) [78]. Intranasal immunization of mice was performed with a combination of PotD and cholera toxin B-subunit. Immunization with PotD resulted in high-titer and specific immune responses in serum and saliva of immunized mice and resulted in significantly reduced nasopharyngeal carriage of both type 19F and type 4.

Mucosal immunization studies in mice with PotD, sortase (SrtA) or glutamyl tRNA synthetase (Gts) either alone or in combination (rPotD+rGts, rPotD+rSrtA, rPotD+rGts+rSrtA) followed by challenge with type 2 strain [77] were performed. Intranasal immunization with anti-sera against single antigen reduced colonization. Intraperitoneal immunization with combinations of rPotD+rGts, rPotD+rSrtA, rPotD+rGts+rSrtA sera afforded better protection against sepsis, with the triple antigen combination affording the highest protection [77]. Passive immunization with combination of sera against multiple antigens was more efficacious against colonization and invasive infections. Mucosal immunization also afforded protection against intranasal challenge with a type 2 strain, with the highest survival rate for a combination of PotD with Gts or SrtA. Immunization with a combination of PotD, Gts, and SrtA afforded higher protection than immunization with any single antigen. Furthermore, PotD, Gts, and SrtA, or anti-sera of mice immunized with these proteins, could inhibit the adhesion of type 2 strain to A549 human lung epithelial cells. Combination of anti-sera had an additive effect on this inhibition. Stimulation of splenocytes from immunized mice resulted in higher expression IFN-γ, IL-4, IL-10 and IL-17A compared to controls after in vitro stimulation with PotD.

Subcutaneous immunization of mice with PotD followed with subsequent intranasal challenge with a type 6B strain has been reported [79]. Immunization with PotD induced a strong IgG response and a significant production of IFN-γ, IL-2, IL-5 and IL-17 by splenocytes, and increased nitric oxide from peritoneal cells following in vitro stimulation with PotD. Immune sera raised against PotD promotes opsonophagocytosis by murine peritoneal cells, and affords protection against nasopharyngeal colonization [79].

Several pneumococcal proteins conserved across serotypes, such as pneumococcal surface protein A (PspA), are being evaluated for next-generation protein-based vaccines. The protective efficacy of a PspA-PotD fusion protein was evaluated [80]. Mice immunized subcutaneously by PspA-PotD fusion protein compared with immunization with single antigens showed that the chimeric protein elicited high antibody titers and was more immunogenic than individual proteins. Opsonophagocytosis with murine peritoneal cells showed increased phagocytosis of multiple capsule types, with the most pronounced effect on type 3 pneumococci. Immunization with PspA-PotD fusion protein afforded protection against invasive infection and colonization. Reduced carriage correlated with the level of IL-17 produced by splenocytes from immunized mice. These studies demonstrate the efficacy of polyamine transport protein against pneumococcal carriage and invasive disease by itself or in combination with other pneumococcal proteins including PotD chimeric proteins.

## Conclusions

Polyamines are common intracellular metabolites of nearly all cells, spanning all domains of life. Their conservation across a vast diversity of cells suggests critical roles for these compounds in cellular physiology. Multiple functions have been ascribed to polyamines, however, it is their interactions with nucleic acids and other highly negatively-charged moieties inside cells which have attracted the most attention. Because of their affinity for nucleic acids, polyamines are potent effectors of replication, transcription, and translation, and consequently have global effects on gene expression. Intracellular polyamine pools in bacteria are tightly controlled by both de novo synthesis and transport. Some cellular states such as rapid cell division and stress are known to increase polyamine flux, yet the full extent of polyamine regulatory mechanisms is still being defined.

Bacterial pathogens of humans regulate polyamine metabolism by mechanisms shared with other prokaryotic cells. This dynamic state of polyamine flux is emerging as a critical parameter of multiple pathways and physiology, with substantial impact on bacterial pathogenesis. This is no less the case for *Streptococcus pneumoniae,* in which polyamine metabolism is emerging as an important regulator of many functions (Table 2). Polyamine homeostasis impacts central metabolism and has broad effects on pneumococcal physiology important for survival and growth in a host. Further exploration of how polyamines exert their outsized influence on pneumococcal pathogenesis will undoubtedly point to novel approaches for treatment and prevention of pneumococcal disease.

**Table 2:** Functions of polyamines in *Streptococcus pneumoniae*

Strain	Function
WU2	Association with cell wall
WU2, D39	Autolysis
D39	Choline homeostasis
D39	Thermal stress
TIGR4	Regulation of galactose metabolism
TIGR4	Regulation of glycolysis
TIGR4	Regulation of pentose phosphate pathway
TIGR4	Regulation of capsule biosynthesis
TIGR4	Regulation of nucleotide sugar synthesis
TIGR4	Oxidative stress

## Abbreviations

WT: Wild-Type
THY: Todd-Hewitt Yeast extract
CDM: Chemically Defined Medium
CPS: Capsular Polysaccharide
PotD: Substrate binding component of polyamine transporter PotABCD
NESp: Nonencapsulated pneumococcus
PPP: Pentose Phosphate Pathway
